# Survey of Washington clinicians’ willingness to use and preferences related to extreme risk protection orders

**DOI:** 10.1016/j.pmedr.2022.101883

**Published:** 2022-07-05

**Authors:** Emma L. Gause, Kelsey Conrick, Megan Moore, Ali Rowhani-Rahbar, Frederick P. Rivara

**Affiliations:** a Firearm Injury and Policy Research Program, University of Washington, United States; b School of Social Work, University of Washington, United States; c Department of Epidemiology, University of Washington, United States; d Department of Pediatrics, University of Washington, United States

**Keywords:** Extreme risk protection order, ERPO, Firearms, Injury prevention, Clinician

## Abstract

Extreme Risk Protection Orders (ERPOs) temporarily restrict access to firearms if an individual is deemed a significant risk of harm to themselves or others. Some states allow clinicians to initiate ERPO petitions for their patients and a new Justice Department model statute recommends clinicians should be eligible petitioners. Washington clinicians cannot currently file ERPOs independently. This article presents the results of an electronic survey of all actively licensed Washington physicians and advanced registered nurse practitioners in 2021 to gauge clinicians’ familiarity, willingness, barriers, facilitators, and preferences for initiating ERPOs by counselling a patient or patient’s family, contacting law enforcement, or filing independently.

3021 Clinicians responded. 75.2% were not familiar with ERPOs but reported being willing to counsel patients about ERPOs if they encountered a patient at substantial risk of harm to themselves (96%) or others (97%). Counselling was the preferred approach to filing; however, approximately 75% would be willing to file independently if allowed. Lack of knowledge about ERPOs was the most reported barrier and training the most common facilitator for all initiation approaches. Having a trained social worker to refer patients (81.5%), an ERPO liaison to law enforcement (70.9%), or coordinator to assist with filing (71.3%) was highly desired. Survey response rates were: 13.5% for physicians, 17.2% for nurse practitioners.

Washington clinicians are willing to use ERPOs for their patients, but they need training. Counselling was the preferred initiation approach, and there was a strong preference for a social worker or ERPO coordinator to assist in counseling and filing.

## Highlights

• Most Washington clinicians are willing to initiate extreme risk protection orders.
• Counselling the patient or family members is the preferred ERPO initiation approach.
• More training on understanding and using ERPOs is needed and desired by clinicians.
• A social worker or coordinator to assist in counseling/filing would facilitate use.

## 1 Introduction

Firearm-related injury was a leading cause of death in the United States in 2020 across all age groups, and the number one leading cause for youth and young adults. Over 45,000 Americans died from firearms, approximately 124 people per day, with suicide accounting for the majority of firearm-related deaths (Centers for Disease Control and Prevention, 2022). Evidence suggests that access to household firearms increases risk of death by suicide three-fold and death by homicide two-fold (Anglemyer et al., 2014). Thus, restricting firearm access among individuals at high risk might be one of the most individually effective methods of preventing suicide or homicide death, but few interventions exist to do so in a timely, targeted manner.

Many firearm access-related policies seek to restrict new firearm purchases for people with a history of domestic violence perpetration, violent misdemeanors, or involuntary mental health treatment, but these are both over- and under-inclusive in their classifications of prohibited persons in terms of who may be at risk of harm to themselves or others. Additionally, while these policies have shown a reduction in firearm homicides, evidence remains inconclusive for suicide (Siegel et al., 2019, Siegel et al., 2020). Policies focused on restricting new firearm purchases are also not applicable for firearms already in the household. Firearm ownership is common in the United States with approximately 44% of individuals living in a household with a firearm in 2020 Saad (2020). Extreme Risk Protection Order (ERPO) laws address these policy gaps by temporarily restricting access to firearms by removing existing firearms already in the home as well as prohibiting new purchases for the order’s duration (Swanson, 2021). They are also a more targeted approach as they are initiated for specific individuals as a result of concerning threats or behavior.

ERPO laws have gained favor in recent years as a firearm injury prevention tool with fifteen states passing new ERPO laws since 2018. As of October 2021, nineteen states and the District of Columbia have enacted ERPO laws (Cherney et al., 2019). ERPOs temporarily restrict an individual’s access to firearms if they are deemed to be at significant risk of harm to themselves or others. An ERPO is a civil court order and follows due process. ERPOs start as a petition to a judge who determines whether there is enough evidence of an imminent threat to grant the order. If an ERPO is granted, the individual, or “respondent,” is prohibited from purchasing firearms and any firearms the respondent owns are removed and stored by law enforcement or family members. After the order’s duration, one year in Washington, the firearms can be returned to the respondent (Rowhani-Rahbar et al., 2020).

Previous research on the effectiveness of ERPOs have found promising results suggesting ERPOs are effective tools to prevent firearm suicide deaths. One study evaluating Connecticut and Indiana ERPO laws found law implementation was associated with a 7.6% reduction in Indiana’s firearm suicide rate over ten years, and a reduction of 13.7% in Connecticut after enforcement was increased (Kivisto and Phalen, 2018). A similar study of Connecticut’s law estimated one suicide death may be prevented for every ten to twenty firearm seizures (Swanson et al., 2022). ERPO petitions are also being used with the intent to prevent mass shootings and other firearm assaults when the respondent seemed at substantial risk of harming others (Wintemute et al., 2019, Frattaroli et al., 2020, Rowhani-Rahbar et al., 2020).

ERPO laws differ between states regarding who is allowed to file petitions. In Washington, only law enforcement, family or household members, and dating partners are eligible to initiate an ERPO (Extreme Risk Protection Orders, 2022). Washington clinicians are not currently able to file ERPO petitions independently, but may counsel patients or patients’ families about ERPOs, for example as part of the patient’s care plan, or contact law enforcement to start the petition in cases where harm is deemed imminent – examples of such use have been documented since the passage of Washington’s ERPO law in 2016 (Conrick et al., 2021). Indeed, clinicians may be uniquely positioned to recognize and intervene when they encounter patients at risk of harm before harm occurs (Pallin et al., 2019). For this reason, the Consortium for Risk-Based Firearm Policy and other firearm injury researchers have called for clinicians to be included as eligible ERPO petitioners by law (Extreme Risk Protection Orders, 2020, Swanson et al., 2021). Hawaii, Maryland, and the District of Columbia have already expanded petitioner eligibility to include certain types of medical and mental healthcare clinicians, including physicians and some nurse practitioners (Cherney et al., 2019). The Justice Department’s model ERPO statute also proposes certain clinicians should be eligible petitioners (Extreme Risk Protection Order Model Legislation, 2021), so it is possible eligibility may expand in coming years.

After passage of Maryland’s ERPO law allowing physicians to petition, a sample of 92 Johns Hopkins physicians were surveyed about their opinions regarding filing ERPOs; time to file a petition and concerns that filing an ERPO would negatively affect their patient relationship were the two most commonly identified barriers. Having an ERPO coordinator and more training on ERPOs were to most requested solutions (Frattaroli et al., 2019). More information is needed to understand clinicians preferences for filing ERPOs, (Blackwood and Christopher, 2021) and to ascertain whether these views are shared by clinicians in different settings.

This study surveyed all actively licensed Washington physicians and nurse practitioners about their familiarity, willingness, and preferences regarding ERPOs for patients. Clinicians were asked their opinions about three different ERPO petition initiation approaches: 1). Counseling a patient or patient’s family 2) Contacting law enforcement about an ERPO for a patient, or 3) Filing an ERPO independently. Washington clinicians are allowed to counsel or contact law enforcement, but they are not currently designated as eligible petitioners. Questions related to independent filing were asked to gauge clinicians’ willingness should this option become available. Clinicians were asked separately about their willingness to use ERPOs if they were to encounter a patient who was at substantial risk of harm to themselves or others. Lastly, clinicians were asked to identify potential barriers or facilitators to using ERPOs for their patients.

## 2 Methods

The survey was developed with the following domains: participant demographics, familiarity with ERPOs, willingness to help initiate an ERPO, barriers or facilitators to using ERPOs, and preferences for how to initiate an ERPO. The electronic survey was created in Research Electronic Data Capture (REDCap), a secure web-based data collection tool hosted by the Institute of Translational Health Sciences at the University of Washington (Harris et al., 2009).

A list of all licensed physicians and advanced registered nurse practitioners in Washington and their contact information was obtained through a records request to the Washington Department of Health. Clinicians with an “active” or “military” license and who had a mailing address within Washington or a bordering state were deemed eligible to participate. Washington physicians and nurse practitioners must renew their license every two years and thus the licensing and contact information was not more than two years old. Clinicians without a valid email address were excluded. Returned surveys from clinicians who reported having no interaction with patients were removed from analysis and deemed ineligible.

An emailed survey link was sent to every physician and nurse practitioner who matched the above inclusion and exclusion criteria. A reminder was emailed every eight days to all clinicians who had not yet completed the survey for a total of three reminders. The nurse practitioner survey opened on May 10, 2021, the physician survey on May 12, 2021. Both closed on June 14, 2021. Responses were tracked via REDCap and email to quantify the volume of undelivered emails, clinicians with explicit or implied refusals, and those ineligible to participate (e.g., retired, deceased, terminated, or on extended leave). Records where the clinician left the survey early without answering any of the barrier or facilitator questions were removed from analysis and considered non-responses. Partial responses with minimal missingness were retained and missingness was reported. Response rates were estimated from the American Association for Public Opinion Research (AAPOR) Standard Definitions and compared in sensitivity analyses using various methods to estimate the proportion of unknown cases that are eligible, including the conservative proportional allocation method and applying an external sampling frame from the Washington Medical Commission’s physician demographic census (The American Association for Public Opinion Research, 2016, Smith, 2009, Physician Demographic Census Aggregate Report, 2022).

Descriptive statistics were used to assess response prevalence. Results from a subset of clinical specialties with high patient interaction were analyzed separately since these clinicians may be more likely to practice in a setting that supports directly engaging with patients regarding their behavioral health concerns, including firearms, and thus may be more able to identify qualifying patients to initiate an ERPO. These included: general internal medicine, family medicine, pediatrics, emergency medicine, and psychiatry.

All analyses were performed in R Studio using R Version 4.0.5 (R Core Team, 2021) and statistical packages: dplyr (Wickham et al., 2021), likert (Bryer and Speerschneider, 2016). This study was reviewed and approved by the University of Washington Human Subjects Division.

## 3 Results

23,051 physicians and 8,049 nurse practitioners were eligible to participate and were emailed the survey. The final analysis set contained 2,034 physician responses (1,921 complete) and 987 nurse practitioner responses (940 complete). Considering both complete and partial responses, the conservative response rates were 10.2%-physicians and 13.4%-nurse practitioners. According to the Washington Medical Commission’s 2021 physician demographic census, only 76% of actively licensed physicians were practicing in Washington (Physician Demographic Census Aggregate Report, 2022). When the proportion of unknown cases that were eligible was recalculated to remove the 24% that were not practicing, response rates became 13.5%-physicians and 17.2%-nurse practitioners, assuming a similar ratio for this group. The appendix contains additional response rate details.

Around half of physicians (55.8%) and nurse practitioners (42.4%) had been practicing for more than 10 years (Table 1), and nearly all participants practiced primarily in an urban area. The most common specialties for physicians were general internal medicine followed by family medicine and pediatrics, while for nurse practitioners nearly half were in family medicine or general internal medicine. 2,088 participants worked in a specialty with high patient interaction (69.1%). Survey responses between nurse practitioners and physicians were similar, thus results are presented in aggregate and referred to as “clinicians” for the remainder of this article. The appendix includes results stratified by provider type and specialty.

**Table 1:** Participant Characteristics.

n (%)	Nurse practitioner n = 987	Physician n = 2,034
**Sex^**		
Female	883 (89.5)	1059 (52.1)
Male	104 (10.5)	968 (47.6)

**Years Practicing**		
<5 years	308 (31.3)	490 (24.2)
5–10 years	259 (26.3)	403 (19.9)
11–15 years	131 (13.3)	302 (14.9)
16–20 years	90 (9.1)	211 (10.4)
More than 20 years	197 (20.0)	618 (30.5)

Specialty*		
Anesthesiology	59 (6.0)	125 (6.2)
Emergency medicine or pediatric emergency medicine	48 (4.9)	168 (8.3)
Family medicine	299 (30.3)	360 (17.7)
General internal medicine or internal medicine subspecialty	154 (15.6)	417 (20.6)
Obstetrics and gynecology	76 (7.7)	82 (4.0)
Pediatrics or pediatric subspecialty	96 (9.7)	326 (16.1)
Psychiatry	87 (8.8)	126 (6.2)
Surgery or surgical subspecialty	59 (6.0)	260 (12.8)
Other specialty	109 (11.0)	165 (8.1)

**Facility Setting**		
Rural	78 (8.1)	96 (4.9)
Urban	889 (91.9)	1872 (95.1)

Note: missingness < 5% excluded from table.

^Clinician sex obtained from licensure data which allowed only “male” or “female” responses.

*Some “Other specialty” write in options have been aggregated and combined with existing categories for summary purposes.

Washington clinicians reported commonly seeing patients who may be at substantial risk of harm to themselves or others, particularly clinicians in specialties with high patient interaction (Table 2). Overall, 25.7% of clinicians reported encountering patients whom they believed to be at substantial risk of harm to themselves at least weekly, while 10.1% reported encountering patients whom they believed to be at substantial risk of harm to others with the same frequency. This proportion rose to 32.8% and 12.7% among clinicians in specialties with high patient interaction for harm to self and harm to others respectively.

**Table 2:** Clinician patient context and firearm injury prevention familiarity.

n(%)	All Survey Participants N = 3,021	Specialties with High Patient Interaction^ n = 2088
**Encounter patients at substantial risk of harm to themselves**		
Daily	253 (8.4)	235 (11.3)
Weekly	524 (17.3)	450 (21.6)
Monthly	638 (21.1)	494 (23.7)
A few times a year	1300 (43.0)	769 (36.8)
Never	305 (10.1)	139 (6.7)

**Encounter patients at substantial risk of harm to others**		
Daily	92 (3.0)	83 (4.0)
Weekly	216 (7.1)	182 (8.7)
Monthly	324 (10.7)	254 (12.2)
A few times a year	1408 (46.6)	964 (46.2)
Never	976 (32.3)	601 (28.8)

**Familiarity with Extreme Risk Protection Orders**		
Very familiar	41 (1.4)	38 (1.8)
Somewhat familiar	214 (7.1)	167 (8.0)
A little familiar	487 (16.1)	363 (17.4)
Not at all familiar	2272 (75.2)	1515 (72.6)

**Currently talk to patients about firearms (multi-select)**		
Yes, always	228 (7.5)	212 (10.2)
Yes, when I am worried about suicidal ideation	1216 (40.3)	1064 (51.0)
Yes, when I am worried about homicidal ideation	989 (32.7)	874 (41.9)
Yes, if the patient brings up firearms	719 (23.8)	603 (28.9)
No, not usually	1059 (35.1)	610 (29.2)
No, this is not an appropriate topic for me to discuss	270 (8.9)	73 (3.5)
Other	144 (4.8)	119 (5.7)
Prefer not to say	18 (0.6)	14 (0.7)
Missing	167 (5.5)	95 (4.5)

Note: missingness < 5% excluded from table.

^Specialties include: Emergency medicine or pediatric emergency medicine, family medicine, general internal medicine or internal medicine subspecialty, pediatrics or pediatric subspecialty, and psychiatry.

A minority of participants were familiar with ERPO laws and how they could be used to prevent injury (Table 2). Only 8.4% reported being “very” or “somewhat familiar” while 75.2% reported having no familiarity at all. Even fewer clinicians reported engaging with ERPOs in their practice in the previous year; 4.4% had counseled a patient or patient’s family or referred a patient to a social worker about seeking an ERPO, and 1.4% had contacted law enforcement about an ERPO. However, many clinicians reported they already discussed firearms with patients; 10.2% of high patient interaction specialists reported always talking to their patients about firearms, while 41.9% and 51.0% discussed firearms if they were worried about homicidal or suicidal ideation respectively. 27.3% of high patient interaction specialists reported having conversations with patients about temporarily storing a firearm outside the home, suggesting that these specialists may be well-suited to incorporating ERPOs into their practice. Notably, 8.9% of participants overall and 3.5% among high patient interaction specialists felt discussing firearms with patients was not appropriate.

**Fig. 1:**
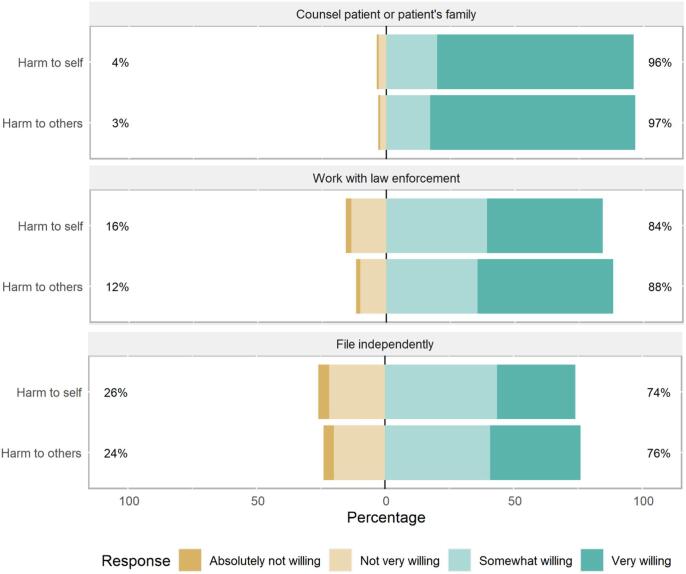
Willingness to engage in ERPOs by method and patient risk.

After a brief description of ERPOs, clinicians were asked about their willingness to initiate ERPOs by counselling a patient or family, working with law enforcement, or filing independently. Results were similar when clinicians were asked about when they believed a patient was at substantial risk of harm to themselves, or others (Fig. 1). The majority of participants were willing to use all ERPO initiation approaches. When a patient may be at risk of self-harm, participants were willing to counsel a patient or patient’s family (98%), work with law enforcement (84%), and file an ERPO independently if this option was provided to them (74%). These proportions were slightly higher if a patient was a potential harm to others.

The most identified barrier for all three initiation approaches was a lack of knowledge about ERPOs – 83.2% of participants reported this as a barrier for counselling, 71.0% for working with law enforcement, and 79.6% for filing an ERPO independently (Table 3). Being unsure about what type of behavior or threats would qualify a patient for an ERPO was also commonly reported. A substantial proportion of participants were worried that using ERPOs might negatively affect their relationship with patients, particularly if initiating through law enforcement (40.3%). Participants with high patient interaction specialties were slightly more likely to report concerns about involving law enforcement. Specific barriers for filing independently also appeared with 57.1% of participants concerned about attending a hearing at the courthouse and 56.3% saying there was not time to file independently.

**Table 3:** Barriers and facilitators to engaging with ERPOs by initiation approach.

n (%)	Counselling patient or patient’s family	Working with law enforcement	Filing independently
**Barriers**			
Lack of knowledge about the ERPO process	2513 (83.2)	2144 (71.0)	2406 (79.6)
Unsure what types of behaviors or threats would qualify for an ERPO	1729 (57.2)	1517 (50.2)	1690 (55.9)
Not enough time during patient encounter	1130 (37.4)	1089 (36.0)	1702 (56.3)
It may negatively affect my relationship with the patient	1032 (34.2)	1217 (40.3)	1066 (35.3)
Current reimbursement structures do not incentivize this	282 (9.3)	275 (9.1)	468 (15.5)
I have concerns about involving the court system	726 (24.0)	878 (29.1)	806 (26.7)
I have concerns about involving law enforcement	–	1338 (44.3)	–
Unable to attend hearing at courthouse	–	–	1726 (57.1)
Other	184 (6.1)	117 (3.9)	115 (3.8)
There are no barriers	157 (5.2)	164 (5.4)	69 (2.3)
I don't think clinicians should do this	24 (0.8)	42 (1.4)	77 (2.5)

**Facilitators**			
Training for clinicians about ERPOs	2319 (76.8)	2095 (69.3)	2138 (70.8)
Being able to consult with a legal expert	1138 (37.7)	1225 (40.5)	1552 (51.4)
If there were a social worker or liaison to refer patient or patient's family to	2463 (81.5)	–	–
Having an informational pamphlet to give to patient or patient's family	1777 (58.8)	–	–
If there were a law enforcement ERPO liaison to work with	–	1938 (64.2)	–
If there were a crisis worker ERPO liaison to work with	–	2143 (70.9)	–
If there were an ERPO coordinator to help me with the paperwork	–	–	2155 (71.3)
Being able to attend the court hearing remotely	–	–	1489 (49.3)
Other	67 (2.2)	68 (2.3)	83 (2.7)
Nothing would make me feel more willing	79 (2.6)	147 (4.9)	312 (10.3)

Note: Missingness < 5% excluded from table.

Not all response barrier and facilitator options were appropriate for each ERPO type. The dashed cells above represent that options that were not offered for that ERPO initiation approach.

The most popular facilitators were aligned with reported barriers – one of the most reported facilitators across all initiation approaches was training for clinicians about ERPOs (Table 3). Participants with high patient interaction specialties were slightly more likely to request training. Having a specific ERPO coordinator or liaison was also highly desired; 81.5% of participants reported they would like to refer patients to a social worker for ERPO counseling, 70.9% reported having a crisis worker liaison when involving law enforcement would be helpful, and 71.3% reported they would like to have an ERPO coordinator to help with paperwork if they were to file the ERPO themselves.

**Fig. 2:**
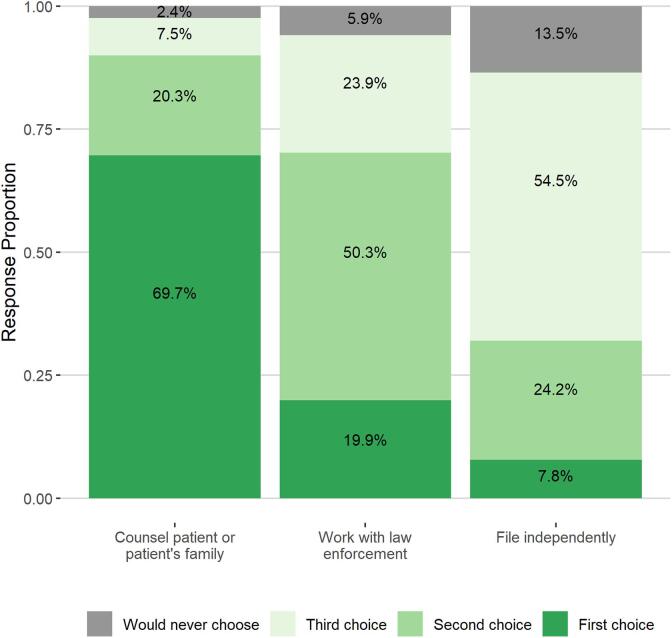
Clinician preferences for engaging with ERPOs.

Lastly, surveyed clinicians ranked their preferences for the three ERPO initiation approaches, allowing them to indicate they would never choose an approach. Over two-thirds (69.7%) of participants reported counselling a patient or patient’s family would be their first choice, while 19.9% preferred working with law enforcement and 7.8% preferred filing independently (Fig. 2). Filing independently was also the most common option participants reported they would never choose (13.5%).

## 4 Discussion

While most Washington clinicians who participated in the survey would be willing to use ERPOs, the majority were not familiar with ERPOs or how they might be used for injury prevention. Over half of clinicians in specialties with high patient interaction reported encountering patients who may be at substantial risk of harm to themselves at least monthly, and one-quarter encounter patients who might be at substantial risk of harm to others monthly suggesting ERPOs may be a useful tool in a clinical setting. More education for clinicians about ERPOs appears to be necessary to increase utilization of ERPOs in a clinical setting and may be a more useful step in preventing firearm injury than simply expanding petitioner eligibility to include healthcare providers. In fact, clinicians identified a lack of knowledge of ERPOs as the most common potential barrier that might prevent them from initiating an ERPO for all ERPO initiation approaches. Fittingly, more training was one of the most requested facilitators for promoting ERPO usage among clinicians.

This study builds on previous findings of ERPO use in a clinical setting by explicitly asking about clinicians’ preferences for how to engage with ERPOs in three ways and their unique barriers and facilitators. Washington clinicians surveyed overwhelmingly preferred counselling a patient or patient’s family of the three initiation approaches. Being able to refer patients to a social worker to counsel was also highly desired. Having help or guidance when using ERPOs was a common theme, either being able to refer patients for counselling, working with a dedicated ERPO liaison, or having someone to file paperwork if clinicians could file independently. Investing resources to provide training to all clinicians about ERPOs generally and perhaps designating ERPO specialists who could follow through the ERPO process with patients or law enforcement could have the most impact for promoting use of ERPOs among clinicians. Since high patient interaction specialists reported a higher frequency or seeing patients at substantial risk of harm, and over half were already discussing firearms with their patients, targeting training and support for clinicians working in psychiatry, pediatrics, internal, emergency, and family medicine may be most successful.

Results from Washington clinicians regarding filing ERPOs independently were similar to responses from a small survey of 92 physicians at Johns Hopkins Hospital in Baltimore (Frattaroli et al., 2019). The Baltimore survey asked specifically about filing an ERPO petition independently as physicians are eligible petitioners in Maryland. The largest barrier identified was time to complete paperwork or attend a hearing, which was also commonly reported among Washington participants. The most promising tools were the same among Maryland and Washington clinicians: training on ERPOs for healthcare providers and a coordinator to complete and follow through with the petition. Similarly, a study of firearm-injury prevention among emergency department physicians across the United States found about half of emergency department physicians reported being comfortable discussing firearms with their high-risk patients, but over 70% said they needed more training on what to do if they believed their patient may be at risk of a firearm injury (Farcy et al., 2021). Training on ERPOs specifically, and firearm safety generally is needed and desired by clinicians.

While clinicians may be in a position to recognize patients at high risk of harm to themselves or others who may also have access to a firearm, few interventions are available to curtail this risk. Clinicians can counsel patients and families on firearm safety and safe storage, exercise their “duty to warn” by informing law enforcement of a potential threat, suggest a voluntary hold or commit a patient to inpatient psychiatric care, and now can work to initiate ERPOs to immediately and temporarily restrict patients’ access to firearms (Shultz et al., 2020). Each intervention involves tradeoffs between safeguarding the wellbeing of the patient and the patient’s autonomy to varying degrees (Shultz et al., 2020). Even though counselling was the preferred method of initiating ERPOs among Washington participants, when voluntary measures like counselling are not fruitful it is essential for clinicians to have a tool to prevent injury. Most Washington clinicians surveyed have a preference for counselling but are still willing to pursue ERPOs for their patients outside of the clinical encounter by contacting law enforcement if they are concerned about imminent harm.

Although proceeding with patient cooperation is preferable, contacting law enforcement remains an option for circumstances where clinicians do not feel comfortable discussing an ERPO, or when the patient or family does not seem receptive to such a discussion. However, just under half of the Washington participants had concerns about involving law enforcement and reported this may discourage them from contacting law enforcement to initiate an ERPO. It is important to note that ERPOs may eventually involve law enforcement, regardless of whether they are the petitioner, since law enforcement is tasked with removing firearms from the ERPO respondent’s possession (Swanson et al., 2021). Equity concerns have been raised as to whether life-saving ERPOs contribute to the perpetration of injustice through the unequal application of ERPOs between racial groups as a result of implicit bias and systemic racism (Swanson, 2020). Even though ERPOs are civil and not criminal court orders, they still involve bringing potentially vulnerable individuals into contact with law enforcement. A recent study in Washington of ERPOs initiated by a health professional contacting an eligible petitioner found in three of twenty-four cases, a criminal charge was filed for the event that initiated the ERPO, and in two cases criminal charges were filed for violation of the ERPO (Conrick et al., 2021). More research should be done to monitor the implementation of ERPOs with a focus on racial justice as well as injury prevention.

To the authors’ knowledge this is the largest survey of clinicians about ERPOs with 3021 responses, but response rates were low. Surveys of clinicians generally have response rates approximately ten percentage points lower than those of the general public (Flanigan et al., 2008). The proportions of physician participants who specialize in internal medicine, psychiatry, obstetrics and gynecology, surgery, and anesthesiology were roughly equivalent to the overall proportion for the state according to the 2021 Washington physician demographic census (Physician Demographic Census Aggregate Report, 2022). However, physicians who returned the survey were more likely to specialize in emergency medicine, family medicine, and pediatrics – specialties with the most opportunity to engage with ERPOs for their patients. Females were more likely to participate than males among both physicians (52.1% of participants were female compared to 41.1% overall) and nurse practitioners (89.5% participants, 84.3% overall). It should be noted firearms can be a contentious and politically charged issue and the decision to respond or not respond to the survey may have been influenced by clinicians’ individual firearm beliefs or personal ownership.

## 5 Conclusions

ERPOs are an evidence-based firearm-injury prevention tool clinicians can implement to prevent harm before it takes place. Washington physicians and nurse practitioners are willing to use ERPOs to prevent firearm-related injuries among patients but need more training about when and how to initiate these discussions with patients or law enforcement. ERPOs are one tool in the toolbox of interventions to prevent firearm injury in a clinical setting and ERPO consideration and firearm access screening should be implemented both in a primary care and emergency setting when patients may be at substantial risk of harm to themselves or others. Promoting the use of ERPOs and providing support to clinicians for counselling patients or working with law enforcement may have more impact in promoting ERPOs than simply expanding petitioner eligibility to include clinicians.

## 6 Role of the funding source

This study was supported by funds from the State of Washington to the Firearm Injury & Policy Research Program. The funder had no role in the design, conduct, analysis, interpretation, or write up of this study, nor the decision to submit for publication.

### CRediT authorship contribution statement

**Emma L. Gause:** Conceptualization, Methodology, Formal analysis, Visualization, Writing – original draft, Writing – review & editing. **Kelsey Conrick:** Conceptualization, Methodology, Writing – review & editing. **Megan Moore:** Conceptualization, Writing – review & editing. **Ali Rowhani-Rahbar:** Conceptualization, Writing – review & editing. **Frederick P. Rivara:** Conceptualization, Writing – review & editing, Supervision.

## Declaration of Competing Interest

The authors declare that they have no known competing financial interests or personal relationships that could have appeared to influence the work reported in this paper.
