# Supplementary material for: Survey of Washington clinicians’ willingness to use and preferences related to extreme risk protection orders
Source: Prev Med Rep. 2022 Jul 5;28:101883. doi: 10.1016/j.pmedr.2022.101883 (PMC9287355; doi:10.1016/j.pmedr.2022.101883)
Supplement: Supplementary data 1 [file mmc1.docx]

**APPENDIX:**

**Table 1: American Association for Public Opinion Research Response Rate Calculation**

|  | **AAPOR**  **Disposition**  **Codes** | **Physician Survey** | **ARNP Survey** |
| --- | --- | --- | --- |
| **DETAILED RESPONSES BY CATEGORY** | | | |
| **Interview (Category 1)** |  |  |  |
| Complete | 1.0/1.10 | 1921 | 940 |
| Partial | 1.2000 | 113 | 47 |
|  |  |  |  |
| **Eligible, non-interview (Category 2)** | 2.0000 |  |  |
| Refusal | 2.1100 |  |  |
| Known-respondent refusal | 2.1120 | 18 | 10 |
| Logged on to survey, did not complete any item | 2.1121 | 1 | 2 |
| Break off/ Implicit refusal | 2.1200 | 224 | 140 |
| Non-contact | 2.2000 |  |  |
| Respondent unavailable during field period | 2.2600 | 34 | 12 |
| Other, non-refusals | 2.9000 | 3 | 0 |
|  |  |  |  |
| **Unknown eligibility, non-interview (Category 3)** | 3.0000 |  |  |
| Unknown about address | 3.1000 | 18561 | 6610 |
| Unknown mail returned undelivered | 3.3000 | 1809 | 174 |
| Returned with forwarding information | 3.4000 | 15 | 6 |
|  |  |  |  |
| **Not eligible (Category 4)** | 4.0000 |  |  |
| Out of sample - other strata than originally coded | 4.1000 | 330 | 55 |
| Not eligible - duplicate listing | 4.8100 | 22 | 53 |
|  |  |  |  |
| **SUMMARY OF RESPONSE CATEGORIES** | | | |
| **Total sample used** | | 23051 | 8049 |
| I = Complete Interviews | | 1921 | 940 |
| P = Partial Interviews | | 113 | 47 |
| R = Refusal and break off | | 243 | 152 |
| NC = Non Contact | | 34 | 12 |
| O = Other | | 3 | 0 |
| **Calculating e:**  *e is the estimated proportion of cases of unknown eligibility that are eligible. This estimate is based on the proportion of eligible units among all units in the sample for which a definitive determination of status was obtained (a conservative estimate)* | | 0.868 | 0.914 |
| U = Unknown Eligibility | | 20,385 | 6,790 |
| **Response Rate 1** | | 0.085 | 0.118 |
| I/((I+P)+(R+NC+O)+U) | |  |  |
| **Response Rate 2** | | 0.090 | 0.124 |
| (I+P)/((I+P)+(R+NC+O)+U) | |  |  |
| **Response Rate 3** | | 0.096 | 0.128 |
| I/((I+P)+(R+NC+O)+e(U)) | |  |  |
| **Response Rate 4** | | 0.102 | 0.134 |
| (I+P)/((I+P)+(R+NC+O)+e(U)) | |  |  |
| **Cooperation Rate 1** | | 0.843 | 0.825 |
| I/((I+P)+R+O) | |  |  |
| **Cooperation Rate 2** | | 0.892 | 0.867 |
| (I+P)/((I+P)+R+O)) | |  |  |
| **Cooperation Rate 3** | | 0.844 | 0.825 |
| I/((I+P)+R) | |  |  |
| **Cooperation Rate 4** | | 0.893 | 0.867 |
| (I+P)/((I+P)+R) | |  |  |
| **Refusal Rate 1** | | 0.011 | 0.019 |
| R/((I+P)+(R+NC+O)+U) | |  |  |
| **Refusal Rate 2** | | 0.012 | 0.021 |
| R/((I+P)+(R+NC+O)+e(U)) | |  |  |
| **Refusal Rate 3** | | 0.105 | 0.132 |
| R/((I+P)+(R+NC+O)) | |  |  |
| **Contact Rate 1** | |  | 0.143 |
| ((I+P)+R+O)/((I+P)+(R+NC+O)+U) | | 0.100 |  |
| **Contact Rate 2** | |  | 0.155 |
| ((I+P)+R+O)/((I+P)+(R+NC+O)+e(U)) | | 0.114 |  |
| **Contact Rate 3** | |  | 0.990 |
| ((I+P)+R+O)/((I+P)+(R+NC+O)) | | 0.985 |  |
| **RECALCULATING THE PROPORTION OF UNKNOWNS THAT ARE ELIGIBLE** | | | |
| **Calculating e2:**  *e is the estimated proportion of cases of unknown eligibility that are eligible. This estimate is based on the proportion of eligible units among all units in the sample for which a definitive determination of status was obtained (a conservative estimate), and subtracting the proportion of licensed physicians who were not currently practicing in Washington (24%)* | | 0.628 | 0.674 |
| **Modified Response Rate 3** | | 0.127 | 0.164 |
| I/((I+P)+(R+NC+O)+e2(U)) | |  |  |
| **Modified Response Rate 4** | | 0.135 | 0.172 |
| (I+P)/((I+P)+(R+NC+O)+e2(U)) | |  |  |

**Table 2: Patient context and firearm injury prevention familiarity, stratified by provider type**

| n(%) | **ARNP**  n = 987 | **Physician**  n = 2,034 |
| --- | --- | --- |
| **Encounter patients at substantial risk of harm to themselves** |  |  |
| Daily | 69 (7.0) | 184 (9.0) |
| Weekly | 161 (16.3) | 363 (17.8) |
| Monthly | 201 (20.4) | 437 (21.5) |
| A few times a year | 438 (44.4) | 862 (42.4) |
| Never | 117 (11.9) | 188 (9.2) |
| **Encounter patients at substantial risk of harm to others** |  |  |
| Daily | 30 (3.0) | 62 (3.0) |
| Weekly | 54 (5.5) | 162 (8.0) |
| Monthly | 93 (9.4) | 231 (11.4) |
| A few times a year | 456 (46.2) | 952 (46.8) |
| Never | 353 (35.8) | 623 (30.6) |
| **Familiarity with Extreme Risk Protection Orders** |  |  |
| Very familiar | 11 (1.1) | 30 (1.5) |
| Somewhat familiar | 60 (6.1) | 154 (7.6) |
| A little familiar | 159 (16.1) | 328 (16.1) |
| Not at all familiar | 755 (76.5) | 1517 (74.6) |
| **Currently talk to patients about firearms (multi-select)** |  |  |
| Yes, always | 83 (8.4) | 145 (7.1) |
| Yes, when I am worried about suicidal ideation | 399 (40.4) | 817 (40.2) |
| Yes, when I am worried about homicidal ideation | 328 (33.2) | 661 (32.5) |
| Yes, if the patient brings up firearms | 243 (24.6) | 476 (23.4) |
| No, not usually | 334 (33.8) | 725 (35.6) |
| No, this is not an appropriate topic for me to discuss | 90 (9.1) | 180 (8.8) |
| Other | 45 (4.6) | 99 (4.9) |
| Prefer not to say | 8 (0.8) | 10 (0.5) |
| Missing | 49 (5.0) | 118 (5.8) |

Note: Missingness <5% excluded from table

**Table 3: Willingness to use ERPOs, stratified by provider type**

|  | n(%) | **ARNP**  n = 987 | **Physician**  n = 2,034 |
| --- | --- | --- | --- |
| **Counsel patient or patient’s family** | **…when patient is at substantial risk of harm to themselves** |  |  |
|  | Very willing | 733 (74.3) | 1571 (77.2) |
|  | Somewhat willing | 208 (21.1) | 384 (18.9) |
|  | Not very willing | 32 (3.2) | 59 (2.9) |
|  | Absolutely not willing | 10 (1.0) | 14 (0.7) |
|  | **…when patient is at substantial risk of harm to others** |  |  |
|  | Very willing | 768 (77.8) | 1626 (79.9) |
|  | Somewhat willing | 180 (18.2) | 328 (16.1) |
|  | Not very willing | 23 (2.3) | 47 (2.3) |
|  | Absolutely not willing | 9 (0.9) | 16 (0.8) |
| **Work with law enforcement** | **…when patient is at substantial risk of harm to themselves** |  |  |
|  | Very willing | 478 (48.4) | 868 (42.7) |
|  | Somewhat willing | 349 (35.4) | 819 (40.3) |
|  | Not very willing | 125 (12.7) | 283 (13.9) |
|  | Absolutely not willing | 21 (2.1) | 43 (2.1) |
|  | **…when patient is at substantial risk of harm to others** |  |  |
|  | Very willing | 546 (55.3) | 1019 (50.1) |
|  | Somewhat willing | 315 (31.9) | 737 (36.2) |
|  | Not very willing | 92 (9.3) | 207 (10.2) |
|  | Absolutely not willing | 17 (1.7) | 33 (1.6) |
| **File independently** | **…when patient is at substantial risk of harm to themselves** |  |  |
|  | Very willing | 332 (33.6) | 560 (27.5) |
|  | Somewhat willing | 410 (41.5) | 863 (42.4) |
|  | Not very willing | 176 (17.8) | 459 (22.6) |
|  | Absolutely not willing | 42 (4.3) | 85 (4.2) |
|  | **…when patient is at substantial risk of harm to others** |  |  |
|  | Very willing | 377 (38.2) | 648 (31.9) |
|  | Somewhat willing | 380 (38.5) | 810 (39.8) |
|  | Not very willing | 161 (16.3) | 418 (20.6) |
|  | Absolutely not willing | 38 (3.9) | 80 (3.9) |

Note: Missingness <5% excluded from table

**Table 4: Willingness to use ERPOs, specific to providers in high patient interaction specialties**

|  | n(%) | **All Survey Participants**  N=3,021 | **Specialties with High Patient Interaction^**  n=2,088 |
| --- | --- | --- | --- |
| **Counsel patient or patient’s family** | **…when patient is at substantial risk of harm to themselves** |  |  |
|  | Very willing | 2304 (76.3) | 1648 (78.9) |
|  | Somewhat willing | 592 (19.6) | 365 (17.5) |
|  | Not very willing | 91 (3.0) | 57 (2.7) |
|  | Absolutely not willing | 24 (0.8) | 10 (0.5) |
|  | **…when patient is at substantial risk of harm to others** |  |  |
|  | Very willing | 2394 (79.2) | 1705 (81.7) |
|  | Somewhat willing | 508 (16.8) | 313 (15.0) |
|  | Not very willing | 70 (2.3) | 42 (2.0) |
|  | Absolutely not willing | 25 (0.8) | 11 (0.5) |
| **Work with law enforcement** | **…when patient is at substantial risk of harm to themselves** |  |  |
|  | Very willing | 1346 (44.6) | 942 (45.1) |
|  | Somewhat willing | 1168 (38.7) | 831 (39.8) |
|  | Not very willing | 408 (13.5) | 256 (12.3) |
|  | Absolutely not willing | 64 (2.1) | 37 (1.8) |
|  | **…when patient is at substantial risk of harm to others** |  |  |
|  | Very willing | 1565 (51.8) | 1100 (52.7) |
|  | Somewhat willing | 1052 (34.8) | 743 (35.6) |
|  | Not very willing | 299 (9.9) | 184 (8.8) |
|  | Absolutely not willing | 50 (1.7) | 27 (1.3) |
| **File independently** | **…when patient is at substantial risk of harm to themselves** |  |  |
|  | Very willing | 892 (29.5) | 648 (31.0) |
|  | Somewhat willing | 1273 (42.1) | 907 (43.4) |
|  | Not very willing | 635 (21.0) | 410 (19.6) |
|  | Absolutely not willing | 127 (4.2) | 67 (3.2) |
|  | **…when patient is at substantial risk of harm to others** |  |  |
|  | Very willing | 1025 (33.9) | 735 (35.2) |
|  | Somewhat willing | 1190 (39.4) | 863 (41.3) |
|  | Not very willing | 579 (19.2) | 362 (17.3) |
|  | Absolutely not willing | 118 (3.9) | 61 (2.9) |

Note: Missingness <5% excluded from table

^Specialties include: Emergency medicine or pediatric emergency medicine, family medicine, general internal medicine or internal medicine subspecialty, pediatrics or pediatric subspecialty, and psychiatry
